# Strategies for eliciting and synthesizing evidence for guidelines in rare diseases

**DOI:** 10.1186/s12874-019-0713-0

**Published:** 2019-03-28

**Authors:** Menaka Pai, Cindy H. T. Yeung, Elie A. Akl, Andrea Darzi, Christopher Hillis, Kimberly Legault, Joerg J. Meerpohl, Nancy Santesso, Domenica Taruscio, Madeleine Verhovsek, Holger J. Schünemann, Alfonso Iorio

**Affiliations:** 1 McMaster University, Hamilton, Canada; 2 Hamilton Regional Laboratory Medicine Program, Hamilton, Canada; 3 American University of Beirut GRADE Center, Beirut, Lebanon; 4 Cochrane Germany, Medical Center, University of Freiburg, Freiburg, Germany; 5 National Center for Rare Diseases, Istituto Superiore di Sanità, Rome, Italy; 6 Cochrane Canada, Hamilton, Canada; 7 Hamilton General Hospital, Room 1-270A, 237 Barton Street East, Hamilton, ON L8L 2X2 Canada

**Keywords:** Clinical practice guideline, Evidence-based medicine, Health care quality, access, and evaluation, Rare diseases

## Abstract

**Background:**

Rare diseases are a global public health priority. Though each disease is rare, when taken together the thousands of known rare diseases cause significant morbidity and mortality, impact quality of life, and confer a social and economic burden on families and communities. These conditions are, by their nature, encountered very infrequently by individual clinicians, who may feel unprepared to address their diagnosis and treatment. Clinical practice guidelines are necessary to support clinical and policy decisions. However, creating guidelines for rare diseases presents specific challenges, including a paucity of high certainty evidence to inform panel recommendations.

**Methods:**

This paper draws from the authors’ experience in the development of clinical practice guidelines for three rare diseases: hemophilia, sickle cell disease, and catastrophic antiphospholipid syndrome.

**Results:**

We have summarized a number of strategies for eliciting and synthesizing evidence that are compatible with the rigorous, internationally accepted standards for guideline development set out by the Grading of Recommendations Assessment, Development and Evaluation (GRADE) system. These strategies include: use of pre-existing and ad hoc qualitative research, use of systematic observation forms, use of registry data, and thoughtful use of indirect evidence. Their use in three real guideline development efforts, as well as their theoretical underpinnings, are discussed. Avenues for future research to improve clinical practice guideline creation for rare diseases – and any disease affected by a relative lack of evidence - are also identified.

**Conclusions:**

Rigorous clinical practice guidelines are needed to improve the care of the millions of people worldwide who suffer from rare diseases. Innovative evidence elicitation and synthesis methods will benefit not only the rare disease community, but also individuals with common diseases who have rare presentations, suffer rare complications, or require nascent therapies. Further refinement and improved uptake of these innovative methods should lead to higher quality clinical practice guidelines in rare diseases.

## Background

### Rare diseases: a Global Health priority

The U.S. National Institutes of Health (NIH) Office of Rare Diseases Research defines a disease as rare if it affects less than 1 in 1500 people, while the European Union defines rare diseases as those affecting less than 1 in 2000 people [1, 2]. Rare diseases frequently cause significant morbidity and mortality, gravely affect quality of life, and confer a social and economic burden on families and communities. Though any one rare disease will affect a small number of people, when taken together, rare diseases affect over 25 million American citizens and 30 million European Union citizens. Moreover, a disease defined as rare in the general population may be endemic to specific populations, conferring disproportionate impact on these communities. Rare diseases challenge traditional health economy perspectives [3]. Many rare diseases have no specific therapies. Those that can be treated with specific orphan drugs are characterized by extremely high costs for the individual, though these amount to a fraction of the total healthcare costs directed towards more common diseases [4, 5]. These considerations, paired with the paucity of available evidence and the inadequacy of accepted metrics to measure quality of life in patients affected by chronic rare diseases (known as the disability paradox), make it challenging to apply standard comparative effectiveness strategies to rare diseases [3, 6].

### Barriers to evidence generation in rare diseases

Most rare diseases are not a focus for policymakers, funders, and researchers in many countries. Pharmaceutical companies may invest significant financial resources in bringing orphan drugs to the market, but often struggle to recover costs through sales [7]. Without Orphan Drug Regulations and cross-sector sponsorship initiatives, there are few economic incentives to develop treatments for rare diseases [8, 9]. And as the number of individuals affected by a rare disease is, by definition, small, a critical mass of people who can advocate for research and development may not exist.

Performing properly sized studies of these diseases, which minimize bias and confounding, is difficult when the number of affected individuals (and potential study participants) for any one disease is low [10]. Adequately powered studies are rarely done because of difficulties in participant recruitment; challenges also include unclear or difficult-to-confirm diagnostic criteria, and lack of reliable patient registries that can support study planning and recruitment [11, 12]. Use of a placebo may not be an option in many rare conditions due to the severe course of the untreated disease, or because patients are unwilling to enroll in studies where they may not receive a badly-needed active treatment option. In order to compensate for low enrollment and/or lack of a comparator, several alternative trial designs (e.g., parallel RCT-cohort trial, sequential design, risk based allocation, hierarchical designs, placebo phase trials, Bayesian designs) have been proposed [13, 14]. However, these innovative types of trials are technically more difficult to design and run than conventional RCTs [15].

Even if a researcher successfully designs and executes a trial, dissemination of results can be challenging. Journal editors may be hesitant to publish studies with a narrow audience, on the assumption that it will have little impact. And much like in common diseases, if a rare disease trial’s findings are negative, publication becomes even more difficult.

When published studies *are* available, they are often heterogeneous. Patients may have widely varying baseline characteristics, interventions and comparators may be used differently, and outcomes and measurement techniques may differ. Patient reported outcomes, such as disease-specific quality of life instruments, may not exist, may not be reported at all, or may be reported with instruments that are neither properly validated nor straightforward to interpret; investigators often rely on an array of surrogate outcomes instead. Heterogeneity in studies can also make aggregating data challenging. The end result of all of these challenges is a slim and inconsistent body of evidence, much of which is low certainty and does not address critically important outcomes.

### Barriers to guideline development for rare diseases

The barriers to guideline development for rare diseases are considerable, spanning from evidence appraisal and synthesis, to issuing recommendations for care, to knowledge translation. Ideally, clinical practice guidelines summarize evidence for patient-important outcomes, and use clear criteria to generate recommendations [16, 17]. In many methodological approaches, high certainty evidence often leads to strong recommendations, whereas low certainty evidence usually leads to conditional, or weaker recommendations. Guideline developers may be unable to generate strong recommendations for diagnosis and/or treatment strategies in rare diseases, due to the lack of high certainty evidence. Guideline users may find these conditional recommendations, which have little evidentiary base, unhelpful or insufficiently directive for frontline clinical care. The ultimate consequence could be suboptimal clinical decisions for individuals with rare diseases. These barriers have previously been described by the RARE-Bestpractices Working Group, a 4 year project (January 2013–December 2016) funded by the European Commission under the FP7 Cooperation Work Programme: Health-2012. The project’s focus was sharing best practices and methodologic knowledge in the clinical management of rare diseases.

Our aim was to use a dedicated framework for guideline development in the field of rare diseases, as proposed by the RARE-Bestpractices Working Group [18]. This framework is intended as an extension of existing guideline development strategies, but has not previously been tested in real rare disease guidelines, Our goal was to determine if: a) the novel strategies for eliciting and synthesizing evidence can be feasibly implemented in guideline development for rare diseases; and b) if the framework helps overcome all or some of the barriers to guideline development described above [18].

## Methods

We piloted the suggested framework in three specific guideline development processes undertaken in three different rare conditions. All three guidelines were coauthored and developed by authors of this report. All three guidelines also explicitly aimed to achieve a double goal: 1) produce a guideline to be used in clinical practice; and 2) implement our framework to overcome expected barriers in guideline issuing in rare diseases. The individuals most focused on the latter goal where the guideline methodologists, whilst the clinical panel members acted as end users trying out and experiencing the impact of our framework.

The first guideline focused on hemophilia, an X-linked bleeding disorder caused by the hereditary lack of a coagulation factor. Blood clotting is severely impaired in hemophilia, resulting in serious bleeding with minimal provocation. Hemophilia A (deficiency of coagulation factor VIII) and hemophilia B (deficiency of coagulation factor IX) are both rare; hemophilia A affects fewer than 1 in 10,000 people and hemophilia B affects fewer than 1 in 50,000 people. In 2016, the National Hemophilia Foundation in the United States partnered with McMaster University in Canada to publish a guideline that explored the optimal model of care delivery, and the optimal composition of the care team, in this rare disease. The guideline used the GRADE (Grading of Recommendations, Assessment, Development and Evaluation) system and the GIN-McMaster guideline development checklist, and adhered to the principles set out by the Institute of Medicine in their 2011 document, “Guidelines We Can Trust.” [19–21] The guideline and its associated studies have been published in the Journal “Haemophilia” and are included in the National Guideline Clearinghouse [22–26].

The second guideline focused on catastrophic antiphospholipid syndrome (CAPS), also known as Asherson’s syndrome. CAPS is a clinical syndrome characterized by the rapid onset of multifocal thrombosis associated with multi-organ failure in patients meeting the serological criteria for antiphospholipid syndrome [27]. The mortality rate for CAPS is about 50%. CAPS is extremely rare, with approximately 500 individuals identified in the published literature since the disorder was first described in 1992 [28, 29]. In 2015, the European RARE-Bestpractices project group partnered with McMaster University to produce guidelines that explored therapeutic and diagnostic questions in this rare disease. The guidelines adhered to GRADE methodology and the GIN-McMaster Guideline Development Checklist [20, 21]. The guideline has been submitted for publication in a peer reviewed journal.

The third guideline focused on sickle cell disease (SCD), an inherited condition in which a mutated form of hemoglobin distorts red blood cells in low oxygen conditions. These crescent shaped “sickle” cells can obstruct the flow of blood in blood vessels, so that oxygen cannot reach nearby tissues. SCD results in anemia, acute painful crises, chronic pain, strokes, and damage to vital organs. The overall prevalence of SCD per 10,000 live births is approximately 30 in African Americans, 0.1 in Caucasian Americans, and 0.3 in Hispanic Americans [30]. In 2016, the European RARE-Bestpractices project (funded by the European Union Commission) organized a group that partnered with the GRADE Centers from American University of Beirut and McMaster Univerisity to produce guidelines exploring diagnostic and therapeutic questions in sickle cell disease [31]. This project’s methods were based on the GRADE and GRADE Adolopment approaches [32, 33]. The project resulted in a manuscript reporting the guideline development process and the resulting recommendations. This manuscript is in the process of journal submission.

All three guidelines were developed using an overarching approach proposed by the GRADE working group. GRADE is a methodological approach that helps guideline developers present summaries of evidence in a structured and standardized way, make transparent judgments about the quality of evidence, and then systematically move towards developing recommendations [34]. GRADE has been adopted by over 100 organizations worldwide, including the World Health Organization [35, 36]. It encompasses not only guideline development for questions about treatment, but also for diagnosis [37, 38]. Most of the applications of GRADE to date have been outside the field of rare diseases, and it is unproven if GRADE can be efficiently applied to rare diseases. For this study, we utilized the RARE-Bestpractice Working Group’s pilot framework for applying GRADE to rare disease guidelines, applying elements of it to all three guidelines and assessing its feasibility and practicability [18]. These rare disease specific elements were as follows:

1. Qualitative research methods can be used to generate evidence on patient values and preferences, equity, acceptability, feasibility, and implementability. These factors are essential to inform Guideline Panel recommendations when using the GRADE system. They are also critical in the guideline implementation phase as they support health policy decision making. However in both rare and common diseases, published evidence on these factors can be difficult to find; it may be necessary to generate it to support guideline development.

2. Expert-based evidence can be systematically solicited through structured observation forms. Despite the paucity of published evidence, there is often a great deal of knowledge about rare diseases – usually from health care providers who cumulatively have many years of real world experience treating patients. Individually, they may not have enough data to publish a peer reviewed study. Furthermore, due to publication bias, data describing ineffective therapies may remain unpublished. However, when data are collected using a structured approach, this “expert-based evidence” becomes invaluable to inform recommendations for clinical practice.

3. Patient registries can be used to supplement the published literature, particularly for questions where scant data were available. Registry data can be used to identify or explore relevant patient subgroups. Registries can also give a snapshot of the impact and natural history of a disease, and the variety and effects of treatments that are used. Registries have the benefit of prospectively capturing patient information; a robust registry, comprehensive in the depth of captured data and inclusive of all known patients, can be equivalent to a high-quality observational study.

4. Finally, indirect evidence can be used. The concept of directness (or indirectness) of evidence is well-established in GRADE, and is used to assess whether the body of evidence used to inform guideline recommendations is directly applicable to the health questions of interest. Sometimes, guideline developers may not be able to find published evidence that directly addresses their clinical questions. In such cases, they can turn to evidence that differs to some degree in terms of population, intervention, comparator, and/or outcome of interest. Indirect evidence becomes particularly valuable in rare diseases. For example, indirect evidence may include extrapolation of data from a population affected by a more common disease that shares some features with the rare disease. (For example, data from epilepsy informing guidelines on tuberous sclerosis.) When the search for applicable/relevant indirect evidence is performed, a specific process to minimize bias can be adopted.

## Results

In all three guidelines, elements of the RARE-Bestpractice Working Group’s pilot framework were successfully used. The specific elements, and our experience with their use, are described below.

### The national Hemophilia Foundation-McMaster guideline on care models in hemophilia

In this guideline, the following elements of the RARE-Bestpractice Working Group’s framework were piloted: indirect evidence; qualitative research; and expert-based evidence.

The NHF-McMaster hemophilia guideline project started with a formal process of priority setting, question generation and prioritization, and identification of patient-important outcomes. Persons with hemophilia (PWH) and their families as well as health care providers were extensively involved in these initial steps. A modified Delphi process was used to finalize guideline questions and outcomes [22, 23].

Due to the paucity of evidence on models of care in hemophilia, the methods group conducted parallel systematic searches for meta-analyses from other chronic diseases to generate indirect evidence [26]. .Ultimately, searches in congestive heart failure, chronic obstructive pulmonary disease, asthma, and diabetes were performed. These diseases shared some features with hemophilia: chronicity, high resource use, involvement over the life span (for asthma and diabetes), and delivery of care via multidisciplinary integrated models [26]. Before the Guideline Panel met (and before they saw the meta-analyzed data for prioritized outcomes), they were asked to judge whether the reviews were “sufficiently direct” that they could inform the care of persons with hemophilia. Based on their summated responses, assessments of indirectness were then incorporated into the evidence profiles. In turn, use of evidence judged to be sufficiently direct provided valuable information on patient preferences, harms, costs and health equity.

There was also scant published information on stakeholder (including patient) experiences and perspectives around health and psychosocial outcomes of importance, acceptability of different models of care, impact of different models of care on health inequities, and feasibility of implementing different models of care. An experienced qualitative researcher on the guideline development team designed and executed a pilot qualitative study to explore these areas [24]. At the outset, stratified purposeful sampling was used to recruit individuals with the following perspectives: people with hemophilia (PWH), parents of PWH aged 18 and under, healthcare providers (hematologists, nurses, physiotherapists, social workers), insurance company representatives, and policy makers. A variety of traditional and social media approaches were used for recruitment. Snowball sampling (where key informants known to members of the research team inform stakeholders about the study) was also used as the study progressed. Participants engaged in semi-structured telephone interviews lasting 25–60 min. Techniques of thematic content analysis were used to identify major and minor themes in the data. The open-ended nature of the interview questions allowed participants to respond according to their own experiences, opinions, and beliefs. Clear themes emerged. Ultimately, this study helped the Guideline Panel learn about care models in 26 hemophilia treatment centers across the United States, and provided insightful information on outcomes of importance to stakeholders, access challenges, impact of care models on health equity, and challenges around acceptability, feasibility, and implementation.

The NHF-McMaster hemophilia guideline adapted surveys developed for a guideline project in Saudi Arabia to systematically capture expert-based clinical observations from the Guideline Panel, as well as a selection of other individuals identified as experts in the field [22, 23, 33]. Respondents were asked, not for their opinions, but for objective information that they could attest to, supported by unpublished data and/or their own observations of the effects of different models of care and care providers on important outcomes. Responses were transparent; the experts were asked to “sign off” on their responses, which were recorded in their entirety. This information was collated and presented to the Guideline Panel.

Patient registries were not used in the NHF-McMaster hemophilia guideline, as the Panel had access to large longitudinal and cross sectional observational studies. However, the guideline ultimately called for Hemophilia Treatment Centers in the United States to reaffirm their commitment to collecting and sharing high quality patient-level data; this enhanced data collection capacity should strengthen the evidence base for future guidelines.

### The McMaster RARE BestPractices guideline on diagnosis and Management of Catastrophic Antiphospholipid Syndrome (CAPS)

In this guideline, the following elements of the RARE-Bestpractice Working Group’s framework were piloted: patient registry data; indirect evidence; and expert-based evidence.

Similar to the NHF-McMaster hemophilia guideline, the initial development stages of the CAPS guideline revealed low certainty to no direct evidence for important outcomes identified by the Panel. Therefore, in addition to the systematic reviews conducted for each guideline question, further methods of data retrieval and evidence creation were used.

The CAPS methods group and Guideline Panel identified the CAPS Registry as a potential source of data to inform the guideline. The CAPS Registry was initiated in 2000 by the European Forum on Antiphospholipid Antibodies to capture diagnosed cases worldwide [28]. Currently, clinical, laboratory, and therapeutic data from over 500 patients are recorded in the registry. The CAPS Registry is the most comprehensive source of patient information in CAPS, with data from both newly diagnosed patients and published case reports. Through on-site, real-time access to the CAPS Registry during evidence generation and panel meeting stages, the Panel was provided with an up to date overview of all indexed CAPS patients. Data from the CAPS Registry (sex, age at time of diagnosis, precipitating factors) informed the Panel on the natural history and impact of the disease. The proportion of deaths associated with each treatment was presented to potentially capture their effects. The CAPS Registry was particularly useful in answering a treatment question regarding the use of rituximab. During the Panel meeting, a search for the mortality of all registry patients who had received rituximab was performed, and compared to the mortality of a contemporaneous cohort of patients not treated with rituximab. A crude odds ratio representing patient mortality with rituximab was presented to the Panel; although rated as very low certainty evidence, it allowed the Panel to consider the potential effects of the drug when previously no information would have been available beyond Panel Member experience.

In contrast to the NHF-McMaster hemophilia guideline, the CAPS methods group did not conduct novel research on patient values and preferences, acceptability, feasibility, or equity. Indirect evidence from other conditions was however used to inform the Panel’s seven outcomes of interest: mortality, permanent organ dysfunction, permanent neurologic deficit, complete recovery, thromboembolic event, and amputation. Recognizing that individuals with CAPS often suffer limb loss and neurologic deficits, the Panel considered patients with diabetes who had limb amputations, and patients with stroke who had neurologic deficits. Health utilities of stroke, acute thromboembolism, and major intracranial bleeds were also considered. Finally, indirect evidence from patients with acute arterial and venous thromboembolism and their bleeding complications (resulting from the use of aspirin, warfarin, and low molecular weight heparins) were considered.

Expert-based evidence was systematically captured from CAPS Panel Members to provide clinical observations for questions around therapy and diagnosis. The collected expert-based evidence was used to inform two key areas. First, the responses around each treatment option provided data on their relative usage in practice. For example, systematically collected observations revealed that only one Panel expert had experience using rituximab. This reflected potential uncertainty around the use of rituximab in wider clinical practice for the Panel to consider. Second, systematically collected observations from Panel Members were discussed and compared to data from systematically reviewed published studies. Panel Members, both spontaneously during the panel meeting and when prompted during an exploratory interview at the end of the guideline development process, stated this was valuable in adopting a standardized and objective way of describing their own practice and experience. Many admitted that their perception of the relevance and direction of the evidence changed when they looked at their experience in a semi-quantitative way. A template of the standardized expert observation form used in the hemophilia and CAPS guidelines is in Appendix A.

### The rare-Bestpractices guideline on sickle cell disease (SCD)

In this guideline, the following elements of the RARE-Bestpractice Working Group’s framework were piloted: qualitative research; and expert-based evidence.

The SCD guideline process started with identifying a source guideline that could be used for the Adolopment process, including to identify subject areas of interest, prioritize questions, and identify relevant studies [33]. After a scoping exercise, the National Heart, Lung and Blood Institute (NHLBI) guidelines on Evidence Based Management of Sickle Cell Disease were selected as the source guideline [39]. The guideline adolopment approach then went through a formal process to prioritize the subject areas tackled by the NIH guideline, prioritize questions for selected areas, and identify patient-important outcomes. For these steps, structured surveys were sent to all Panel Members. Although the Panel prioritized a number of patient important outcomes, due to scarcity of data, the team ultimately focused on the outcomes considered in the NHLBI guidelines.

A methods group, independent from the Guideline Panel, conducted the literature search in three sequential steps for each of the prioritized questions: (1) screening of the NIH guidelines reference list; (2) electronic database search for relevant systematic reviews; and (3) electronic database search for relevant primary studies. Systematic searches were also conducted for values attached to the outcomes of interest, preferences for the interventions under consideration, and resource use associated with the intervention options. The Panel was asked to review the list of identified studies and suggest if any were missed; however no studies reporting direct evidence were identified by the experts beyond what had been captured through the first two search steps. The SCD methods group did not conduct systematic searches for indirect evidence in related diseases.

Panel Members were asked to complete online surveys looking at values and preferences, and resource use related to the interventions of interest. Panelists were also asked to suggest additional published and unpublished information of interest relating to benefits and harms, values and preferences, resource use, acceptability, feasibility, and equity. These additional pieces of information were provided in the form of unstructured communications from Panel Members to the methodology team, and were discussed during the face-to-face Panel Meeting. At their in-person meeting, Panelists were asked to complete a survey soliciting feedback about the guideline development process.

### Ethics approval

The qualitative study conducted as part of the NHF-McMaster hemophilia guideline was submitted to and approved by the Hamilton Integrated Research Ethics Board in Hamilton, Ontario, Canada. Written informed consent was obtained from all participants in the qualitative study. Ethics approval for collection of expert based evidence in all the described guidelines was not deemed necessary, as it involved surveys of guideline panel members, whose ethical treatment and consent to participate in guideline related activities was considered implicit. All other work described in this study involved systematic reviews of anonymized and published data, and thus did not require ethics submission and approval as per the Hamilton Integrated Research Ethics Board in Hamilton, Ontario, Canada.

## Discussion

Rare diseases pose a real challenge to patients, providers, payers and policy makers. Many of these conditions have no satisfactory therapeutic options, while others have very expensive therapeutic options with relatively little evidence to support them [2, 40]. Health care practitioners and policy makers often have little experience with rare conditions. Thus, clinical practice guidelines which summarize the available evidence and provide recommendations developed by experts in a structured and transparent manner are potentially helpful in supporting decision making at various levels.

Our study – in which we applied a framework for evidence synthesis, allowing for the flexible and transparent use of indirect, expert-based, and ad hoc newly generated evidence – successfully addressed some of the “evidence gaps” encountered when developing three target rare disease guidelines. These “gaps” are common to guideline development for many rare diseases. Guidelines in common diseases frequently rely on comparative evidence of treatment effects (generated when two or more interventions, or an intervention and a placebo, are compared). This reliance on comparative studies is often not practical for rare disease guidelines. Indeed, there is often only one treatment for any given rare disease, or there is only very low certainty evidence for the effect of different treatments.

A major strength of our study is that it shows how, in the absence of disease-specific comparative studies, valuable evidence can still be retrieved and successfully used. Systematic reviews of evidence from similar, more common diseases can provide valuable comparative information to Guideline Panels. Qualitative research has proven to be a viable data source for values and preferences, acceptability, feasibility, and equity [41]. These important aspects are underreported in both rare and common diseases. Our study suggests that patient registries are a feasible source of information for guideline developers as well. Registries can capture long term longitudinal data that are difficult to capture in conventional experimental study designs due to ethical, regulatory and financial constraints. Registries may be of particular use in rare diseases, many of which are chronic, and affect individuals over the life span. Registries are considered key instruments for developing rare disease clinical research, enhancing patient care and health planning, and improving social, economic and quality-of-life outcomes. Indeed, it is usually the case that no single institution, and in many cases no single country, has sufficient data on a given rare disease that can be applied broadly to clinical and translational research. However, the fragmentation and heterogeneity of existing registries, which are often the result of spontaneous initiatives, limit the general applicability of their observations [42]. The use of registries in guideline development is subject to other limitations. Ideally, registries must be designed to collect information on patient-important outcomes. Registries must also be set up to capture data that are amenable to pooling in order to achieve a sufficient sample size for key outcomes. This requires significant foresight as the registry is launched, ongoing efforts to maintain recruitment, and assurance that the technology to securely share data is maintained. In the CAPS guideline, many analyses could not be performed because of missing variables that would have controlled for confounding. The guideline development group could therefore provide only crude estimates and unadjusted analyses. Another concern with using registry data is duplication of data from published studies, since populations with rare disease populations are small. For example, data from published studies of CAPS patients were likely incorporated into the CAPS Registry, as part of the registry recruitment strategy is periodic canvassing of the literature to identify and recruit cases. In these circumstances, it may be important to inform the Panel that analyses from the registry could potentially be based on duplicate data already presented in the studies retrieved from the systematic review. Furthermore, use of registries almost inevitably requires involvement of the creators of the registry, which can lead to important intellectual conflicts of interest.

Another strength of our study is its confirmation that unpublished non-experimental data (including observational data and qualitative evidence) can be elicited and collected in such a way to be usable by Guideline Panels. Systematically collected evidence from field experts can provide this information; further, “going to the source” may overcome dissemination bias, albeit at the risk of introducing selection bias (which must be carefully monitored).

Either of the two approaches indicated above can overcome the tendency of guideline developers to focus only on randomized controlled trials and large observational studies to inform their work, which may not be possible outside the field of common diseases. As users demand high-quality guidelines for rare diseases, it will be important to incorporate non-experimental and non-comparative data collected in a systematic and transparent fashion.

There were clear limitations to our work, and to the RARE-Bestpractice Working Group’s framework. Qualitative research did provide valuable information in the NHF-McMaster guideline. However, in general, published qualitative data can be difficult to find in rare diseases – either because these studies are not conducted, or because they are not published. Guideline developers can conduct their own qualitative studies (as the NHF-McMaster group did), but this takes time and resources. Qualitative research may have complemented the limited quantitative evidence base in the CAPS guideline. In particular, it may have provided insight into patient perspectives, which are not reported in the literature. However, identifying patients to interview was a major limitation to conducting a qualitative study of CAPS; the number of patients diagnosed with this rare condition every year is low, the presentation is precipitous and often fulminant, and the mortality rate is close to 50%. In such circumstances, patient organizations may be able to assist with identifying patient perspectives. Partnering with a patient organization ultimately helped the CAPS guideline development team recruit a patient representative for the Guideline Panel. When published qualitative studies are available, there is still little consensus in how to rate the quality of evidence. GRADE-CERQual (Confidence in the Evidence from Reviews of Qualitative research), a GRADE project group, has proposed that an assessment of confidence for individual review findings from qualitative evidence syntheses be based on four components: methodological limitations of the incorporated studies, relevance of the studies to the review question, coherence of the review finding, and adequacy of data supporting the review finding [43]. This is an active area of study, but is currently a major constraint on the utility of qualitative evidence in guideline development.

Finally, our study identified limitations to the use of expert-based evidence. Guideline developers have been advised to help Panel Members identify the evidence underlying their opinions, and judge the quality of that evidence [44]. However, expert-based evidence can be challenging to incorporate into guidelines. We maintain that it should be collected transparently and systematically, and subjected to the same level of Guideline Panel appraisal as other evidence. This evidence can be subject to bias if it tilts from objectively collected data into subjectively developed opinion. Experts should be encouraged to provide statements in advance, after systematically having reviewed cases they have managed, to provide the most objective information. This would provide Panels with unique data gained from years of clinical experience. Experts should also be required to sign off on their statements, which may increase its trustworthiness.

Further research into how novel methods of evidence creation would support the guideline update process is also needed. For example, if a guideline for a rare disease relies heavily on evidence from a more common disease with a rapidly evolving knowledge base, the rare disease guideline may require more frequent updating through reviews of indirect evidence. Guideline developers must also be able to efficiently respond to changes in the knowledge base that may impact guideline recommendations, but can only be detected by ongoing qualitative research (for example, shifts in patient values over time). Finally, it is clear that non-experimental and non-comparative forms of evidence can play a valuable role in guideline creation. Several groups, including AID (Appraising and Including Different Knowledge in Guideline Development) and GRADE-CERQual are actively exploring this concept.

## Conclusion

After theoretically exploring the barriers to issuing guidelines on rare diseases, we proposed an operational approach consistent with GRADE [18]. We have now applied this approach in three specific rare conditions [22]. Our work confirms that priority setting, question generation and prioritization, and outcome identification and ranking, all pursued with extensive involvement of patients and experts are possible in the development of rare disease guidelines, and the process can parallel that in more common diseases. Furthermore, we have come to a number of conclusions: We have concluded that intentional and systematic gathering of (unpublished) observational evidence from experts is a more efficient and transparent alternative to informal “around the table discussion” by the Guideline Panel. We have also concluded that it is feasible to use indirect evidence from other diseases and access a patient registry of the target disease to complement published low certainty evidence. Finally, we have established the feasibility of qualitative research as an important way to glean information about values and preferences, acceptability, feasibility, and health equity – all key factors to consider when making practice recommendations.

All three rare disease guidelines in this study maintained a clear link between quality of evidence and the strength of the ultimate recommendations, as specified by the GRADE Working Group framework. Rigorous clinical practice guidelines are needed to improve the care of the millions of people worldwide who suffer from rare diseases. We recommend that systematic processes must be used to create evidence-based practice guidelines that are useful to patients, clinicians, researchers, industry, and policy makers in the rare disease community. However, these processes must also be dynamic and responsive to the unique challenge presented by rare diseases: a dearth of high certainty evidence. Innovative evidence elicitation and synthesis methods will benefit not only the rare disease community, but also individuals with common diseases who have rare presentations, suffer rare complications, or require nascent therapies. Further refinement and improved uptake of these innovative methods should lead to higher quality clinical practice guidelines in rare diseases.

## Abbreviations

AID: Appraising and Including Different Knowledge in Guideline Development
CAPS: Catastrophic antiphospholipid syndrome
CERQual: Confidence in the Evidence from Reviews of Qualitative research
GRADE: Grading of Recommendations, Assessment, Development and Evaluation
NHLBI: National Heart, Lung and Blood Institute
PWH: Persons with hemophilia
SCD: Sickle cell disease
